# The effect of R547, a cyclin-dependent kinase inhibitor, on hepatocellular carcinoma cell death

**DOI:** 10.3906/biy-1907-3

**Published:** 2020-02-17

**Authors:** Betül HACIOĞLU*, Gökhan KUŞ, Hatice Mehtap KUTLU, Selda KABADERE

**Affiliations:** 1 Department of Physiology, Faculty of Medicine, Eskişehir Osmangazi University, Eskişehir Turkey; 2 Department of Health Programs, Open Faculty, Anadolu University, Eskişehir Turkey; 3 Department of Biology, Faculty of Science, Eskişehir Technical University, Eskişehir Turkey

**Keywords:** R547, hepatocellular carcinoma, Hep G2, H-4-II-E, apoptosis, flow cytometry, microscopy

## Abstract

Hepatocellular carcinoma (HCC) is the third main cause of cancer-related death. Cyclin-dependent kinases (CDKs) and their cyclin partners regulate the cell cycle. Since inhibition of CDKs gives some guiding ideas for cancer studies, we aimed to determine the possible effects of R547, a cyclin kinase 1-2-4 inhibitor, on proliferation and apoptotic mechanisms of Hep G2 cells (human) and H-4-II-E cells derived from rat HCC. We determined in vitro survival rates with MTT assay, apoptosis with flow cytometry, morphological changes with confocal microscopy, and ultrastructural changes by transmission electron microscopy. Cisplatin was used as a positive control. After 24 h of culture with 0.1, 1, 10, 50, and 100 µM doses of R547, the corresponding percentages of live Hep G2 cells were 101%, 94%, 93%, 89%, and 79% (P < 0.001), respectively. However, with the same R547 doses the live Hep G2 cell percentages were 92%, 101%, 53.6% (P <0 .01), 47.4% (P < 0.001), and 41% (P < 0.001), respectively, after 48 h. After 24 h of incubation with the same doses of R547, the survival percentages of live rat cells were 90%, 80% (P < 0.01), 63% (P < 0.001), 47% (P < 0.001), and 43% (P < 0.001), respectively. The percentages of surviving H-4-II-E cells were 96%, 85% (P < 0.01), 46% (P < 0.001), 44% (P < 0.001), and 45% (P < 0.01), respectively, after 48 h. Since R547 did not significantly affect Hep G2 cell survival in 24 h, experiments of apoptosis were carried out with H-4-II-E cells. The early apoptotic rates of 38% and 45% (P < 0.05 for both) after applications of 10 and 25 μM R547 (control: 4.1%), respectively, indicated that R547 has an apoptotic effect on H-4-II-E cells in 24 h. The apoptosis morphology at 24 h of treatment was clearly observed with microscopic examinations. According to our results, it is obvious that R547 has antiproliferative action when compared to cisplatin.

## 1. Introduction

Hepatocellular carcinoma (HCC) is one of the most common types of cancer that originates from hepatocytes. It is more common in males over the age of 50 than females. Approximately 80% of patients with HCC are of Asian or African origin. The risk factors for HCC include chronic viral hepatitis B and hepatitis C infections, alcohol addiction, usage of tobacco and tobacco products, and aflatoxin. The lack of symptoms during HCC makes the diagnosis difficult. Since it is an aggressive tumor, the survival period of patients is only a few years. For approximately 70% of patients with HCC, this period is about 5 years (Wang et al., 2012). Treatment of the disease depends on tumor size and stage, and high-grade tumors result in poor prognosis. Surgery is one of the main treatment methods for HCC; on the other hand, nowadays, liver transplantation is the most effective method (Zhang et al., 2007). The suitability of surgical treatment for patients with HCC is about 20% (Zhang et al., 2012). Radiotherapy and chemotherapy are also treatment options that do not give good expected results. Chemotherapy and the effects of the drugs used are typically limited due to hepatic and systemic toxicities. These agents expose patients to various side effects when causing HCC cell death (Kang et al., 2010). Since HCC needs more effective and less toxic drugs, the discovery of new anticancer drugs that stimulate apoptosis is promising.

The cell cycle in multicellular organisms is very important for proliferation, growth, wound healing, and many other biochemical and physiological processes (Funk, 2005). Cyclin-dependent kinases (CDKs) are members of the serine/threonine protein kinase family. They have very important roles in cell cycle regulation and transcription (Sanchez-Martinez et al., 2015). At the G1 phase of the cell cycle, the CDK4/cyclin D and CDK6/cyclin D complexes regulate the termination of the cycle according to the cell signaling, cellular activities, growth and development, and maintenance of homeostasis in eukaryotic cells through the phosphorylation mechanism. The S phase starts with the CDK2/cyclin A complex, and the CDK1/cyclin B complex regulates the G2 phase and the beginning of the mitotic phase (Shackelford, 1999; Peyressatre, 2015).

Apoptosis has become a focal point of hope for the treatment of diseases via the apoptotic mechanism. Apoptosis is generally known as type I programmed cell death that is characterized by cell shrinkage, DNA concentration, fragmentation, membrane blebbing, depolymerization of the cell skeleton, and apoptotic body formation. It shows caspase activation along death receptors or mitochondrial pathways (Xu et al., 2014). Proliferation and death mechanisms in cells have a certain balance and harmony. 

Since R547 inhibits the CDK1/cyclin B, CDK2/cyclin E, and CDK4/cyclin D1 complexes, the cell cycle is stopped at the transition of the G1 and G2 phases. It is known that it reduces the phosphorylation of retinoblastoma (RB) protein by using the RB pathway, stimulates apoptosis, and causes cancer cell death. It is believed that R547 is a promising molecule for solid tumor treatment during phase I studies in vitro and in vivo in various human tumor xenograft models (De Pinto et al., 2006). Since HCC requires more effective and less toxic treatments, we aimed to reveal the effect of R547, a CDK inhibitor in HCC, which has not yet been elucidated. Based on all of this information, we intended to examine an ATP-competitive CDK inhibitor, R547, at various time and dose intervals on the growth and apoptosis of two HCC cell lines.

## 2. Materials and methods

### 2.1. Cell culture and groups

We used human HCC cell line Hep G2 and rat HCC cell line H-4-II-E (ATCC). The cell lines were fed with a mixture of 10% fetal calf serum (FCS), 1% penicillin-streptomycin, and 89% Dulbecco’s modified Eagle’s medium (DMEM, Sigma-Aldrich) in an incubator that had 37 °C temperature, 5% CO2, and 95% O2 hyperoxide conditions (Oztopcu-Vatan et al., 2015). Both cell lines were treated with 0.1, 1, 10, 50, and 100 µM doses of R547 (Cayman) for 24 h and 48 h. DMSO was used as a vehicle. As a positive control, cisplatin (Sigma-Aldrich) at doses of 5, 10, 25, 50, and 100 µM was applied to both cell lines for 24 and 48 h. The control groups contained only feeding medium. 

### 2.2. Cell survival analysis

After numbers of cells were determined with the trypan blue method by using a CEDEX cell counter (Roche Diagnostics International Ltd.), 1 × 104 cells were plated in each well of 96-well plates. R547 and cisplatin as mentioned above were added for 24 and 48 h by appropriate calculations and each experiment was repeated at least three times. After 24 and 48 h of treatments, survival rates of the cells were determined by 3-[4,5-dimethylthiazol-2yl]-diphenyl tetrazolium bromide (MTT) method (Mossmann, 1983). The optical density was read by a spectrophotometer (BioTek Instruments) at 550 nm wavelength and converted to percentage of living cells according to control cells. The formula is as follows: 

(Cell absorbance of the drug in each well × 100) / Absorbance of control cells

### 2.3. Analyzing apoptosis by flow cytometry

Since according to MTT results, the inhibitory effect of R547 was marked only at the higher doses for 24 h, it was decided not to use the Hep G2 cell line in the following experiments.

Accordingly, 1 × 106 H-4-II-E cells were plated in 25-cm2 flasks with doses of 10 and 50 µM R547 or DMSO and incubated for 24 h. The cells were then removed with 0.25% trypsin-EDTA solution (Sigma-Aldrich). The number of cells in 1 mL was determined by trypan blue method. The cell solution was adjusted with binding buffer to be 1 × 106 cells in number according to the kit’s instructions. H-4-II-E cells, treated or not treated with 10 or 50 μM R547 or DMSO for 24 h, were stained with the FITC Annexin V Apoptosis Assay Kit with propidium iodide (PI, Invitrogen), and then 5 µL of FITC-annexin V, 10 µL of PI, and 100 µL of cell solutions were added carefully into 2-mL vials. The vials were incubated for 15 min in the dark at room temperature, allowing them to mix thoroughly and precisely. After incubation, 400 µL of the annexin V binding solution was added to the vials. These stages of the study were carried out by flow cytometry (Acury BD) in the Pharmaceutical Microbiology Laboratory of the Faculty of Pharmacy at Anadolu University, Eskişehir, Turkey. All experiments were repeated three times.

### 2.4. Observing the morphological changes with confocal microscopy

About 150,000 H-4-II-E cells were seeded in each well of 6-well plates in 5 mL of medium and incubated for 24 h for analyzing possible morphological changes caused by R547. After the incubation period, 10 or 50 µM doses of R547 were applied to each well for 24 h. The medium was removed and cells were washed with cold phosphate buffered saline solution (PBS) and then fixed with 2.5% glutaraldehyde in 0.1 M phosphate buffer (pH 7.4). After fixation, the cells were washed again with cold PBS and dyed with 0.1 mg/mL acridine orange and Alexa Fluor 488 Phalloidin. Morphological changes that occurred in the cells were examined by a Leica SP5 laser scanning confocal microscope and photographs were taken at the Plant, Drug, and Scientific Research Center (BİBAM) of Anadolu University, Eskişehir, Turkey.

### 2.5. Examining ultrastructural changes by transmission electron microscopy (TEM) 

To ensure morphological changes in H-4-II-E cells, 1 × 106 cells were seeded in each 25-cm2 flask. After the incubation period of 24 h, the control group was fed with only 5 mL of medium, and the 10 and 50 µM R547 groups were fed with 5 mL of medium containing the calculated doses of drug and incubated for an additional 24 h. Then the cells were fixed with 2.5% glutaraldehyde for 4 to 24 h at 4 °C. The second fixation in 2% osmium tetroxide was performed to link the unit membrane lipid layers and proteins. Dehydration was achieved by passing through a series of 50%, 70%, 90%, and 96% pure ethanol (100%). The cells were then embedded in EPON 812 epoxy. As a final step, the cells were freshly prepared and placed in a recessed medium and allowed to block for 48 h. When the blocks were ready, the sections were stained with a solution containing heavy metal salts such as uranyl acetate and lead acetate for enhancement and staining. After staining, the sections were rolled on porous or film-coated conductive copper sheets or grids. Morphological changes were examined by using TEM (FEI Bio Tween) at BİBAM.

### 2.6. Statistics

The results of MTT assays were analyzed using one-way analysis of variance and then Tukey’s multiple comparison test by using SPSS 15.0 (SPSS Inc., Chicago, IL, USA). P < 0.05 was considered significant. For evaluating the results of flow cytometry analysis, normalization tests and nonparametric Kruskal–Wallis tests were performed. The results of apoptosis were analyzed by Kolmogorov–Smirnov normality test and then nonparametric Kruskal–Wallis test by using SPSS 16.0 for statistical evaluation of vitality, apoptosis, and death values of the cells after R547 treatments. The apoptotic results are presented as percentages of cells.

## 3. Results

### 3.1. Analyzing cell survival by MTT assay

**Figure 1:**
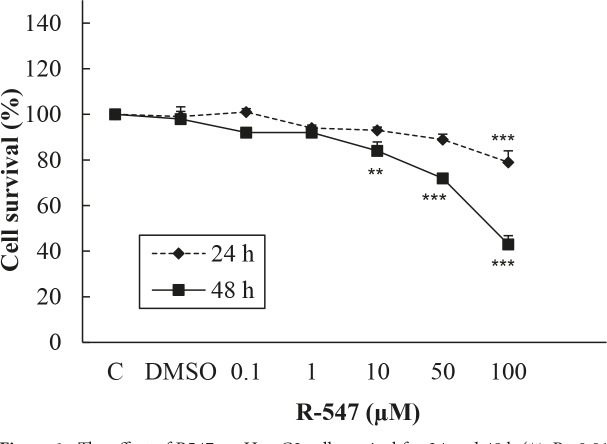
The effect of R547 on Hep G2 cell survival for 24 and 48 h (**: P <0.01 and ***: P < 0.001).

The percentages of living Hep G2 cells treated with R547 at doses of 0.1, 1, 10, 50, and 100 µM for 24 h were found as 101%, 94%, 93%, 89%, and 79% (P < 0.001), respectively. Survival rates of Hep G2 cells treated with the same doses for 48 h were 92%, 101%, 53.6% (P < 0.01), 47.4%, and 41% (P < 0.001), respectively. The IC50 inhibition concentration of R547 for Hep G2 cells was 88 μM for 48 h. There was no change in the survival rates of both cell lines with the highest concentration of DMSO. According to MTT results, the inhibitory effect of R547 was marked only at the highest dose of R547 for 24 h and so it was decided not to use the Hep G2 cell line in the following experiments (Figure 1). 

**Figure 2:**
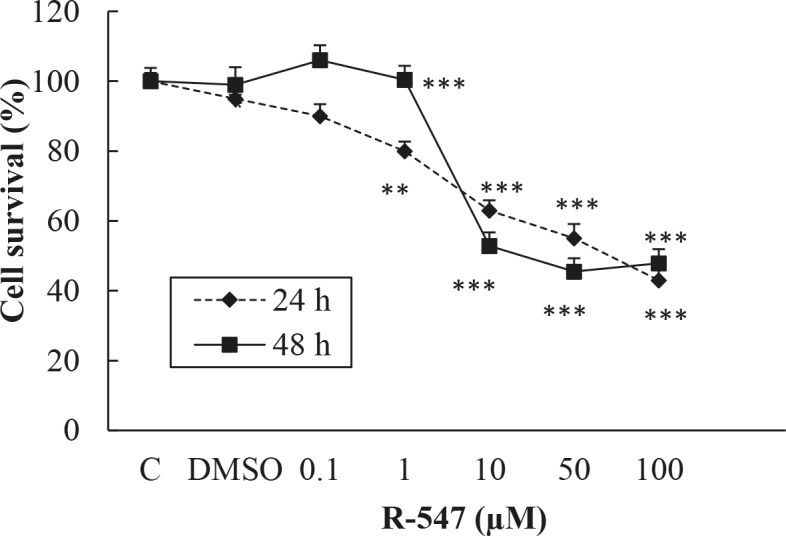
The effects of R547 on H-4-II-E cell viability.

When H-4-II-E cells were treated with the above doses of R547 for 24 h, the percentages of surviving cells were 90%, 80% (P < 0.01), 63%, 47%, and 43% (P < 0.001), respectively. The survival rates of H-4-II-E cells treated with the same R547 doses for 48 h were 96%, 85% (P < 0.01), 46%, 44%, and 45% (P < 0.001), respectively. The IC50 inhibition concentration of R547 for H-4-II-E cells was 66 μM for 24 h, while it was 23 μM for 48 h. Depending on the percentage of cell death rates obtained from 24 and 48 h of treatments, we decided to study H-4-II-E cells for the apoptosis investigation (Figure 2). 

**Figure 3:**
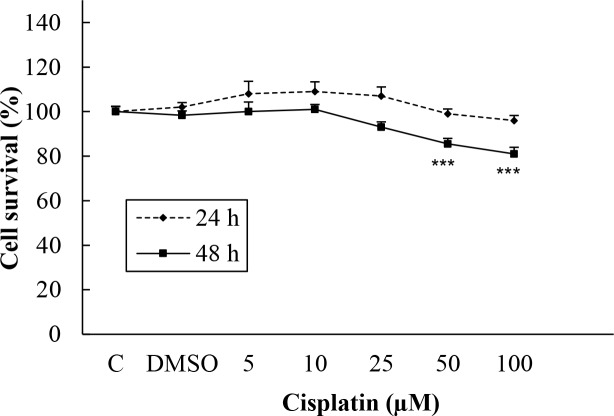
Influence of cisplatin on Hep G2 cell viability.

**Figure 4:**
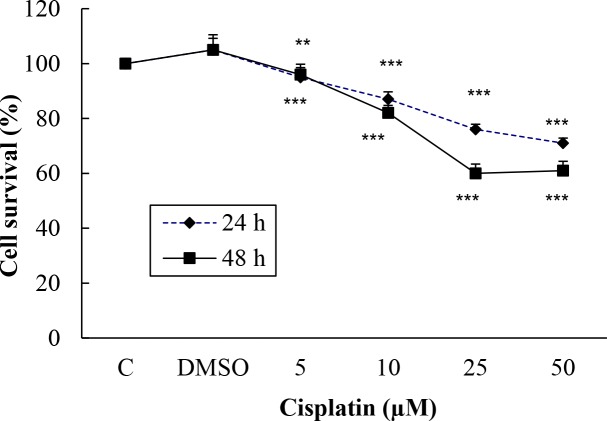
Cisplatin’s effect on H-4-II-E cell survival.

Cisplatin at 5, 10, 25, 50, and 100 µM was used for a positive control for 24 and 48 h for both cell lines. The living Hep G2 cells after 24 h of incubation were calculated as 108%, 109%, 107%, 99%, and 96%, respectively, and for 48 h as 100%, 101%, 93%, 85.5% (P < 0.001), and 81 (P < 0.001) , respectively. When treated with the same doses of cisplatin for 24 h, the percentages of live H-4-II-E cells were calculated as 95%, 87% (P < 0.001), 76% (P < 0.001), 71% (P < 0.001), and 70% (P < 0.001), respectively, and after 48 h as 96%, 82% (P < 0.01), 60% (P < 0.001), 61% (P < 0.001), and 56% (P < 0.001; Figures 3 and 4). 

### 3.2. Results of flow cytometry

**Figure 5:**
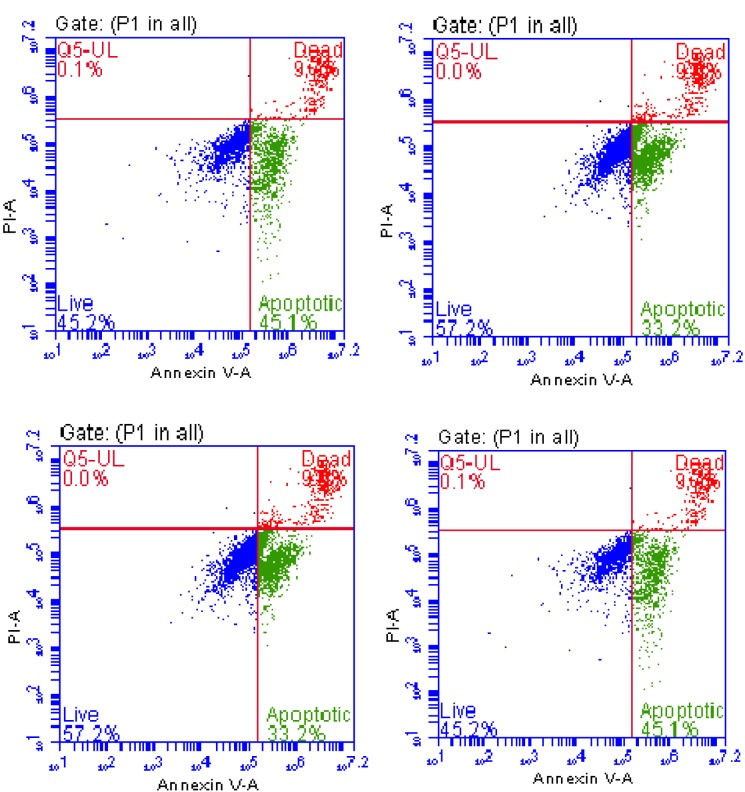
Typical quadrant analysis of annexin V-FITC/PI flow cytometry of H-4-II-E cells that were treated for 24 h with two higher doses of R547 (10 and 50 μM) and DMSO.

The percentages of apoptotic cells were 4.07% in the control group, 2.57% in the DMSO group, 37.8% in the 10 µM R547 group, and 45.4% in the 50 µM R547 group (Figure 5; Table). 

**Table 1:** Proportions of viable, early apoptotic, and late apoptotic/necrotic cells after exposure to 10 and 50 μM doses of R547 or DMSO for 24 h in H-4-II-E cells. *: P < 0.05. The data are expressed as the mean ± SD of the mean. The results are the means of three experiments.

Group	Viable (%)	Early apoptotic (%)	Late apoptotic (%)
Control	92.93 ± 1.51	4.07 ± 0.06	3.20 ± 1.21
DMSO	94.83 ± 1.46	2.57 ± 0.93	2.13 ± 0.51
10 µM	47.03 ± 8.82*	37.80 ± 4.11*	14.87 ± 4.91*
50 µM	45.20 ± 4.20*	45.40 ± 5.86*	9.27 ± 1.72*

### 3.3. Analyzing the morphological changes by confocal microscopy 

**Figure 6:**
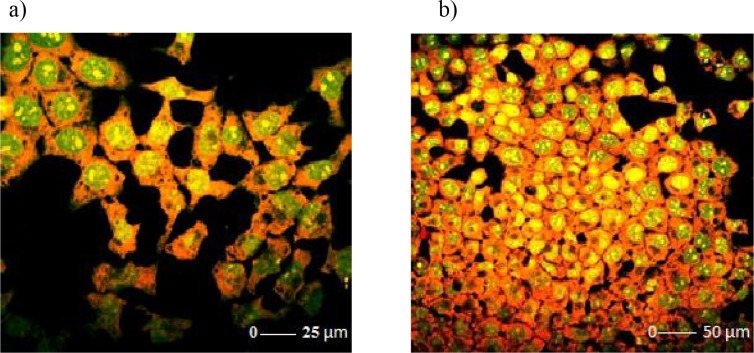
a and b) Confocal microscopic images of the control group of H-4-II-E cells.

H-4-II-E cells, treated with 10 or 50 µM R547 for 24 h, were examined under a confocal microscope and compared with the control. Membranes, nucleus structures, and skeleton structures of the cells were found to be normal in the control group. The cells retained their polygonal structure in their normal state (Figures 6a and 6b).

**Figure 7:**
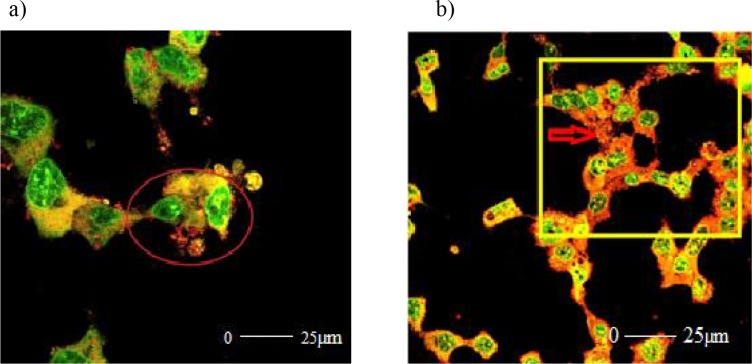
a and b) Confocal microscopic views of 10 μM R547 group. Due to the deformation of the membrane and cell skeleton of the cells, holes that formed in the cell skeleton with budding were observed. Additionally, chromosome degradation was observed in the cell nucleus. The resulting structural defects were indicated by arrows.

In the 10 µM R547 group, the cells lost their morphological integrity. Blebbing and holes occurred as a result of defects in the membranes and skeletons of the cells. The normal structure of the chromosomes was disrupted and fragmented. The cell membrane deformation caused by the outward opening of the cell skeleton with budding out of the cell indicates apoptosis. The structural defects were indicated by circles and squares (Figures 7a and 7b).

**Figure 8:**
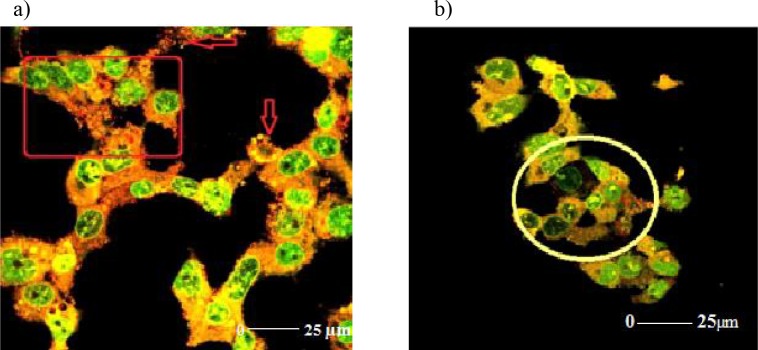
a and b) Confocal microscopic illustrations of 50 μM R547 group. Deformation budding and density of apoptotic bodies in the cell membrane were observed. In addition, hole formation in the cell skeleton and chromosomal deformations in the nucleus were observed. The resulting structural defects are indicated by arrows (a). DNA fragmentation and DNA fractures are shown by circle (b).

In the 50 µM R547 group, the cells were diminished by losing their morphological structures. Blebbing and holes occurred as a result of defects in the cell membrane and skeleton. Normal structure of the chromosomes was disrupted and fragmented, DNA fractures occurred, and apoptotic body formations were observed. The structural defects were indicated by squares and circles (Figures 8a and 8b).

### 3.4. Studying the ultrastructural changes by TEM 

**Figure 9:**
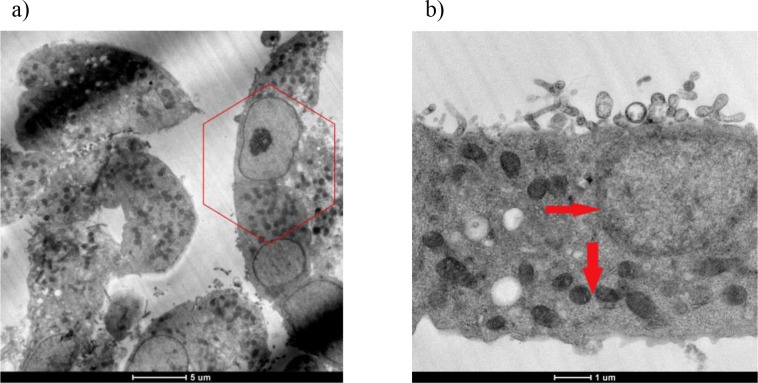
a and b) TEM views of untreated H-4-II-E cells. Hexagon shows normal cell shape (a). Arrows indicate normal cell membrane, shape, and nucleus (b).

In the control group, normal cell membrane, shape, and nucleus were observed and the chromosomes were collected in the nucleus (Figures 9a and 9b).

**Figure 10:**
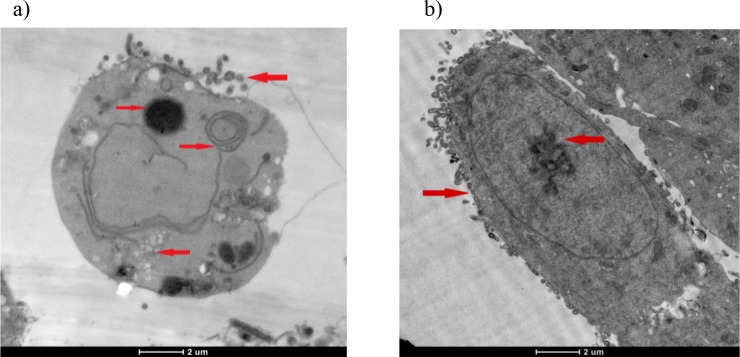
a and b) Photographs of 10 μM R547 group cells from TEM. The cell nucleus was seen to have disappeared. Myelinated sheath formation and autophagic vacuole formation were observed (a). In addition, chromosomes were densified with deformation of the cell nucleus (b).The resulting structural defects were indicated by arrows.

The cell membrane was disrupted and the cell content was dispersed. Along with the deformation of the cell nuclei, chromosomes were concentrated in the 10 µM R547 group. In addition, myelinated sheath formation and autophagic vacuole formation were observed. The structural defects were indicated by arrows (Figures 10a and 10b).

**Figure 11:**
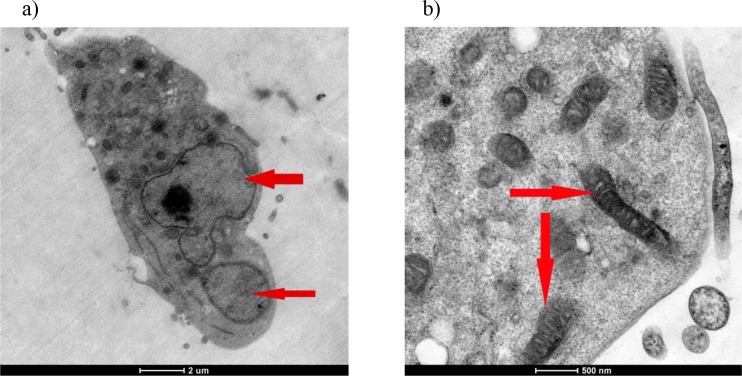
a and b) Structural views of 50 μM R547 group cells from TEM. The cell membrane was deformed and the nucleus of the cell took the shape of a horseshoe. In addition, the mitochondria were swollen and their crystals dispersed (a). Arrows indicate thin and long mitochondria (b).

The structure and content of the cells, mitochondria, and nuclei were impaired in the 50 µM R547 group. The horseshoe shape of the nucleus may suggest that apoptosis occurred (Figure 11a). In addition, the mitochondria were swollen and the contents of the cavities were discharged, and an increase in vacuolization and the deformation of mitochondria were observed with an intense metabolic increase in the cells (Figure 11b). The resulting structural defects were indicated by arrows.

## 4. Discussion 

In the present study, R547 caused only 21% of Hep G2 cell death at the highest dose in 24 h, but it caused 52% and 59% cell death at 50 and 100 µM in 48 h. R547 had greater decreasing efficacy on H-4-II-E cell viability in both 24 and 48 h, as shown in Figure 2. Similarly, in the study by De Pinto et al. (2006), the antiproliferative effects of R547 were examined by MTT assay in colon, breast, lung, prostate, cervix, melanoma, B-cell lymphoma, and osteosarcoma cell lines. Their results indicated that R547 has potent in vitro antiproliferative effects (IC50: approximately 0.05–0.6 µmol/L) on all these cell lines. They also showed that the growth-inhibitory activity is characterized by a cell cycle block at the G1 and G2 phases. In addition, they found that R547 stimulates apoptosis. Additionally, in very recent research, AZD5438 and R547, which are cyclin-dependent kinase inhibitors, showed potential for enhancing the efficacy of daunorubicin-based anticancer therapy (Sorf et al., 2019). Cisplatin is a drug used in chemotherapy during the treatment of various types of cancer by inhibiting DNA synthesis and killing cancer cells (Kishimoto et al., 2016). Also, it was shown that cisplatin, which is used as a positive control, has a weaker effect on viability of Hep G2 and H-4-II-E cells compared to the effect of R547 (Figures 3 and 4). Supporting our results, Kishimoto et al. (2016) did not find any significant effect on cell viability when they treated H-4-II-E and Hep G2 cells with doses of 0.1, 1, and 10 µM cisplatin for 72 h. 

In this study, because R547 had less effect on cell numbers and decreasing activity in Hep-G2 cells in 24 h, we continued the research about apoptosis with rat H-4-II-E cells. We chose 10 and 50 μM doses of R547 because these two doses had the most antiproliferative effects on rat H-4-II-E cells in 24 h. We determined that R547 induces apoptosis of HCC cells dose-dependently in vitro. The percentages of early apoptotic values at 10 and 50 μM were about 38% and 46% for R547, respectively, for 24 h (Table). Our confocal and TEM examinations revealed morphological changes of the cells caused by apoptosis. We observed cell content deterioration, cell shrinkage, DNA fragmentation, cell membrane budding, apoptotic body formation, core condensation, and horseshoe nucleation. It is known that condensation of chromatin and DNA fragmentation are indicators for the determination of apoptosis with TEM (Doonan and Cotter, 2008). There are several studies showing that CDK inhibitors induce apoptosis. For example, Xiao et al. (2014) used paclitaxel for the treatment of malignant tumors, followed by RO3306, which is a CDK1 inhibitor, both separately and in combination on the Hep G2 cell line. They applied paclitaxel at a 0.2 µM dose for 18 h followed by RO3306 at 2 µM for 6 h. According to their study, these two drugs used in combination provided a synergetic inhibition on the cells, moving the cells from mitosis to apoptosis. In another study conducted by Shi et al. (2015), fluspirilene, which is a potential CDK2 inhibitor and is used as an antipsychotic drug, stopped Hep G2 and human HCC cell division in the G1 and S phases depending on dose and time. It revealed changes in CDK2 levels and induced apoptosis. They suggested that fluspirilene can be used in various types of cancer treatments. In another in vitro study, Bollard et al. (2017) mentioned that when used alone or in combination with sorafenib, pablociclib, a CDK4 and CDK6 inhibitor, suppressed proliferation in Hep G2 and other human HCC cell lines depending on dose and time. Zhang et al. (2012) indicated that dihydroartemisinin (DHA), another CDK inhibitor, decreased cell viability by 50% at a dose of 20 µM in 24 h depending on dose. Cho et al. (2010) determined that apoptosis in Hep G2 and different HCC cells was induced via increases in caspase 3 and caspase 9 activation after treatment with 10 and 50 µM doses of xylocydine, a specific CDK inhibitor, for 24 h. Moreover, De Pinto et al. (2006) examined the effects of treatment with R547 on DNA fragmentation. They confirmed that R547 induces apoptosis as measured by DNA fragmentation. All of these indicated results support our findings.

In conclusion, R547 inhibited proliferation of Hep G2 cells at 48 h and H-4-II-E cells at both 24 and 48 h. The same inhibitor caused apoptosis with morphological and ultrastructural changes in H-4-II-E cells in 24 h. Therefore, R547 may have a promising role against HCC as an inhibitor of CDK and an apoptosis-inducing candidate. Further studies are needed to clearly understand the mechanism of R547’s action.
